# Changes in postnatal outcomes following implementation of a family-led postnatal care model: A pre-post intervention study in Ada’a District, Ethiopia

**DOI:** 10.1371/journal.pgph.0006487

**Published:** 2026-10-30

**Authors:** Gadise Bekele Regassa, Della Berhanu, Konjit Wolde, Dedefo Teshite, Lello Amdissa, Walelegn Worku Yallew, Solomon Getachew Alem, Pooja Sripad, Anne Hyre, Lisa Noguchi, Alemayehu Worku

**Affiliations:** 1 Addis Continental Institute of Public Health, Addis Ababa, Ethiopia; 2 Jhpiego Ethiopia, Addis Ababa, Ethiopia; 3 The London School of Hygiene & Tropical Medicine, London, United Kingdom; 4 Jhpiego, Maryland, United States of America; 5 Department of Epidemiology and Biostatistics, School of Public Health, Addis Ababa University, Addis Ababa, Ethiopia; Aga Khan University, PAKISTAN

## Abstract

Postnatal care is crucial for assessing the health of both mother and newborn, yet its coverage remains low in Ethiopia. Timely, high-quality postnatal care, especially within the first week after birth, is essential to reduce maternal and neonatal mortality rates. Family-led postnatal care is an innovative model for reaching postnatal mothers and newborns during the first week of life. Leveraging self-care principles, mothers, with the support from family, perform daily postnatal health checks on themselves and their newborns. This study evaluated the effect of a family-led postnatal assessment intervention on coverage of postnatal checks within seven days of birth. This study used pre- and post-intervention cross-sectional surveys in four health centers. Eligible postnatal mothers who gave birth in the study health centers were interviewed pre- (November 2022-Jan 2023) and post-intervention (February to April 2023) using a structured questionnaire. Bivariate tests and descriptive analyses were used to assess changes in postnatal check coverage over time. Surveys were completed with a total of 119 mother pre-intervention and 110 mother post-intervention. In the pre-intervention period, nine percent (11/119) mothers and 11% (13/119) newborns had a postnatal check between 24 and 72 hours after birth, whereas during the post-intervention period this increased to 96% (105/110) mothers and 96% (105/110) newborns (P < 0.0001). There was a similar increase in the proportion of mothers and newborns having postnatal checks between 73 hours and 7 days (3% vs. 96%, P < 0.0001). Compared to pre-intervention, a larger proportion of mothers detected a maternal danger sign during the post-intervention period (6.7% vs 18.2%, P < 0.008). Family-led postnatal care is a promising self-care model that increased postnatal checks for mothers and newborns who would not otherwise have received care. Evaluating this model in other settings with more rigorous design is recommended. ClinicalTrials.gov (NCT05563974), first posted on 3^rd^ October 2022.

## Introduction

Postnatal care (PNC) is defined by the World Health Organization (WHO) as care given to the mother and her newborn immediately after the birth of the placenta and for the first 42 days after [1]. To ensure maternal and newborn wellbeing, WHO recommends that all postnatal mothers have at least four postnatal checks: within 24 hours after birth, between 24–72 hours, between 72 hours and seven days, and at 42 days [2]. However, despite its importance, PNC remains the most neglected period along the continuum of maternal and newborn care [3], with 49% of maternal deaths occurring on the first day after childbirth and 25% of deaths occurring between days two and seven [4]. Similarly, half of neonatal deaths occur in the first day of life, and nearly three quarters of all neonatal deaths occur within the first week of life [5,6]. Ensuring regular high-quality postnatal visits, particularly within the first week after birth, is paramount to reduce maternal and neonatal morality rate [4,7–11].

The federal government of Ethiopia has a target to increase coverage of early postnatal care within two days from 34% to 76%, with the aim of decreasing the maternal mortality ratio from 401 to 279 per 100,000 live births and neonatal mortality from 33 per 1,000 live births to 21 per 1,000 live births by 2017 Ethiopian fiscal year (2024/25) [6]. According to the Ethiopian Demographic and Health Survey (EDHS) 2016, only 17% of mothers and 13.9% of newborns receive at least one postnatal care service from a health institution in the first 48 hours after birth [12]. Per the national Community Based Newborn Care guidelines, health extension workers should conduct home visits for all postnatal mothers in their communities [13,14]. However, these home visits are not occurring due to many reasons, including health extension worker workload, lack of birth notification to health care providers, poor supervision, inadequate drug supply, distance, and topography [14–16]. At the same time, families generally do not seek PNC at health facilities due to their perceptions of being healthy, lack of awareness of the service, poor experiences with negative attitudes of health care providers, and lack of information [17].

The WHO defines self-care as “the ability of individuals, families and communities to promote health, prevent disease, maintain health and cope with illness and disability with or without the support of a health worker” [18]. Self-care interventions are tools that support self-care. These include evidence-based, high-quality drugs, devices, diagnostics and/or digital interventions that can be provided fully or partially outside formal health services and be used with or without a health worker.

The Ethiopian Ministry of Health has put forward a major strategic initiative to introduce and implement “self-care” initiatives [6]. A guideline has been developed to support the introduction and implementation of self-care interventions for improving maternal and newborn health [19]. The COVID-19 pandemic has generated ample evidence on self-care in both general health and maternal health care contexts [18,20–23]. Many self-care interventions have been implemented with support from the health system at initial stages of use and uptake [22,23]. These studies have showed promising results indicating the possible use and impact of innovative self-care interventions on maternal and neonatal morbidity and mortality.

The design of intervention tested in this study is based on key insights form human-centered design (HCD) activities in the local context. These HCD activities revealed that mothers and newborns were expected to stay home during the first 42 days after birth, particularly the first ten days. Furthermore, in the postnatal period, mothers most trusted their husbands and immediate family members, religious leaders, and neighbors when seeking care/advice or making decisions. Postnatal mothers also felt they were expected to “prove” themselves as good mothers and feared not knowing how to care for their newborn, or how to recognize problems. Postnatal mothers and their families tended to seek care from health facilities as a last resort, when they were in dire need [24], opening the door for a potential culturally sensitive bridge between the home and facility contexts for postnatal assessments.

Family-led postnatal care (FPNC) is an innovative model for reaching postnatal mothers and newborns with key PNC services during the first week of life that leverages self-care principles. The objective of the current study was to evaluate potential changes in selected postnatal care-related outcomes following implementation of a family-led postnatal care intervention on coverage of postnatal checks within seven days of delivery for both mothers and newborns.

### Program description

The FPNC model consists of the following components (Fig 1): Prior to discharge from the health center, a postnatal assessment and counseling is conducted by the health center staff, guided by a visual checklist. A health center staff invites family members into the postnatal room to observe the following: how the physical assessment is conducted; use of the blood pressure machine and infrared thermometer; postnatal counseling; and the postnatal checklist. Following the checklist, the health center staff assess for severe headache, vaginal bleeding, breast conditions, blood pressure, temperature, and swelling in the face and lower extremities. In the newborn, the health center staff checks if the newborn appears to be feeling well, breastfeeding and burping, as well as skin color, umbilical cord conditions, breathing pattern, temperature, and vaccination status.

**Fig 1:**
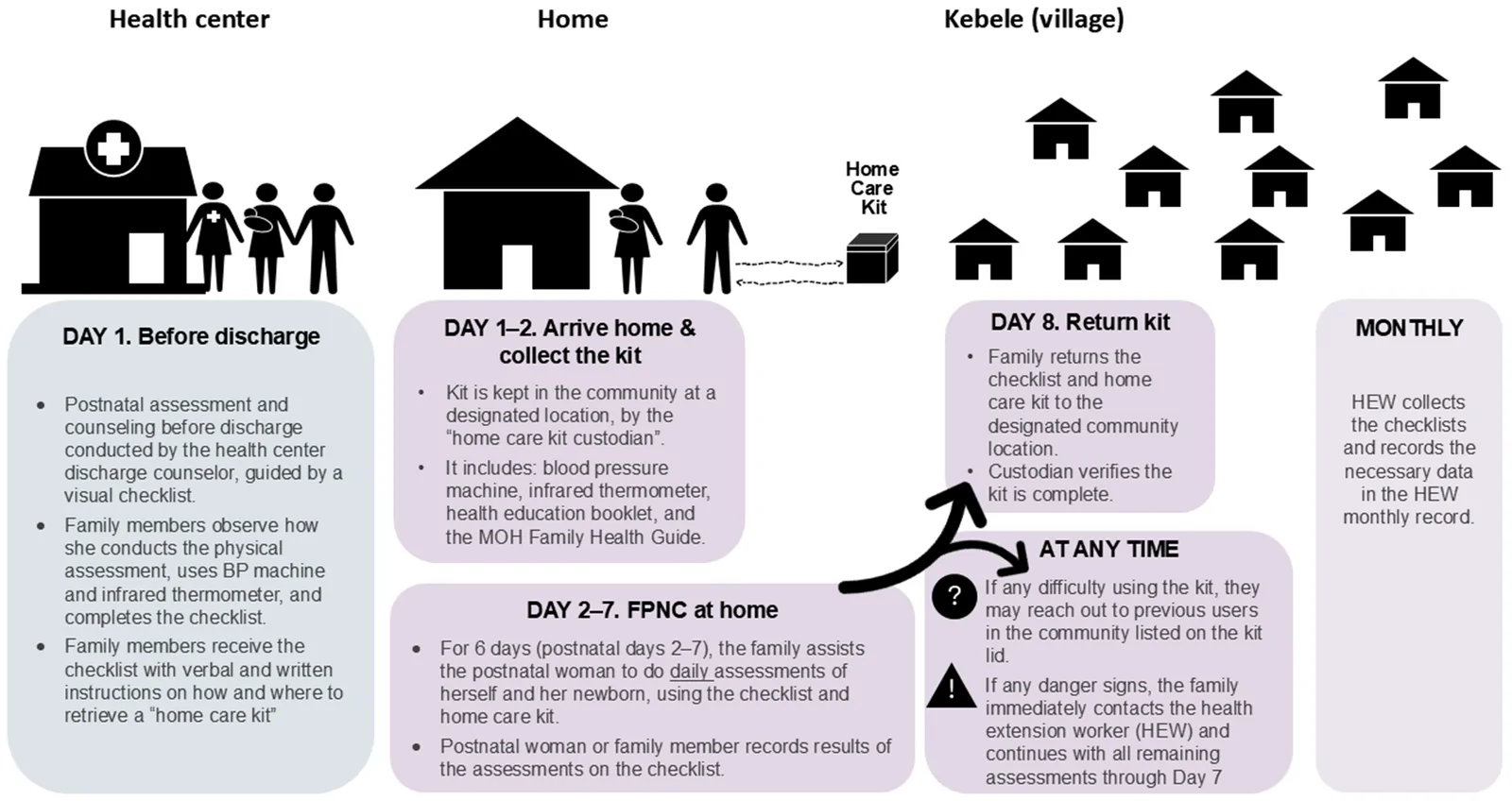
Summary of the FPNC intervention implementation procedures, Ada’a, Oromia, 2023.

Upon discharge, the health center staff gives the checklist to the family with verbal and written instructions on how and where to retrieve a “homecare kit” (HCK) that includes a blood pressure machine, infrared thermometer, and a health education booklet.

Three homecare kits are placed in homes of community among members and community leaders identified by health extension workers to serve as homecare kit “custodians”. After arriving home, a family member retrieves the HCK from the designated custodian, who records the check-out date and location in a register. For six days (postnatal days 2–7), the postnatal mother, with the assistance from family members, conducts daily assessments of herself and her newborn, using the checklist and contents of the HCK. If a family has difficulty using the devices, they may reach out to other families who have previously used the HCK in the community in addition to the HCK custodians and HEWs. A log on the lid of the HCK indicates who else in the community has used the HCK along with their contact information.

The postnatal mother or family member records the results of the assessments on the checklist. If the assessment reveals any danger signs, the checklist instructs the family to immediately contact a health extension worker and to continue with the remaining assessments up to the seventh day. Around the eighth day after delivery, the family returns the checklist to the custodian, who verifies the availability and functionality of the equipment and records its return in the register. The custodian asks the family for permission to list their contact information on the HCK log in case other families need guidance on how to use the devices. If the family member grants permission, the information is recorded on the HCK log. At least monthly, the health extension worker collects the completed checklists and records the assessments in the Integrated Maternal Newborn Child Health Card at the health post [25].

## Methods

### Ethical statement

Ethical approval was obtained from the Johns Hopkins Bloomberg School of Public Health Institutional Review Board (IRB No. 21096) and the Addis Continental Institute of Public Health Institutional Ethical Review Board (IRB No. 0029). Participation in the FPNC study was entirely voluntary. All postnatal mothers identified in the study who met eligibility criteria were offered enrollment. In the post-intervention period, all postnatal mothers who wanted the enhanced approach to discharge assessment and counseling received it, regardless of their decision to enroll into the study. Study procedures, anticipated risks or discomfort, and potential benefits of the study were described to the potential participants. Informed verbal consent was undertaken with each participant upon completion of the discharge assessment and counseling. Data collectors served as witnesses to the consent process and documented it by signing the consent forms. This procedure was approved by both institutional review boards. Confidentiality was maintained to the maximum standard during all stages of study implementation.

### Study setting

Primary level healthcare in Ethiopia is provided by a health center (where a majority of facility-based deliveries take place) and approximately five catchment health posts, where two health extension workers provide curative and preventative services. The study was conducted in four health centers and their catchment *kebeles* (the lowest administrative unit in Ethiopia) in Ada’a district of Oromia Region, Ethiopia. The population of Ada’a is 204,847 with 7,109 annual births. At the time of the study, Ada’a district had 5 health centers, 22 *kebeles*, 22 health posts and 37 health extension workers during the study period. One of five health centers was excluded as it was part of the conceptualization, design, and testing of the FPNC model.

### Study design

This study used quantitative pre- and post- intervention cross sectional surveys. The pre-intervention survey was conducted from November 14, 2022 to Jan 22, 2023 and the post-intervention survey from February 6 to April 9, 2023. Intervention was provided after the pre-intervention data collection was completed.

### Study participants

Mothers who delivered at the four participating health centers were approached to participate in the study. Mothers who were within three days of birth during discharge from the health center, intended to remain within the catchment area of the health center for the first week after birth, had family members who were willing to participate in the study, and were willing and able to provide consent were included in the study.

### Sample size and sampling

The sample size was calculated using the 2016 EDHS coverage of PNC within 24 hours of 17% [12]. With a desired increase PNC coverage to 45% due to the intervention, a 5% level of significance, 80% power, a design effect of 2.0, and a non-response rate of 10%, the minimum sample size required to detect the assumed difference was 109 postnatal mothers at both pre-intervention and post-intervention each, for a total of 218 postnatal mothers. Postnatal mothers who delivered at the study health centers were included sequentially until the sample size was fulfilled.

### Data collection procedures

Training was provided to field data collectors and supervisors on the study objectives, procedures and ethics. A detailed survey manual with extensive standard operating procedures was prepared and used in the training, piloting, and survey itself. The training also included proper use of tablets for data entry at the point of collection.

Health center staff who were involved in the postnatal discharge process at the four selected study health centers screened mothers for participation in the FPNC study. Before discharge from the study health center, the health center staff asked screened mothers if they were interested in speaking with FPNC study staff to learn more about the study. If the mother expressed interest, she was referred to the FPNC study staff. The study staff member who was on site approached all mothers and their accompanying family members to assess further eligibility, explain the study procedures, answer any of their questions, and obtain informed consent. If eligible and consented, study staff obtained contact information from the mother/family member. Study staff communicated the contact information for the local study enumerator. Data collectors assigned to the respective *kebeles* interviewed these mothers on the eighth postnatal day at their home.

### Outcome measurement

Postnatal mothers were interviewed using a structured questionnaire to collect information on basic socio-demographic data (age, educational status, and marital status). The outcome measurements were completed based on mothers’ report in the pre-intervention period and extraction from the postnatal checklist in the post-intervention period. In addition, the following information on the primary and secondary outcomes were collected.

Primary outcomes:

- Proportion of mothers and newborns who had a postnatal check within 24 hours, 24–72 hours, and 73 hours–seven days;
- Proportion of mothers and newborns who assessed key PNC components within 24 hours, 24–72 hours, and 73 hours–seven days (self-report in the pre-intervention and from checklists provided to families in the post-intervention); and
- Average number of days any PNC assessed (mothers and newborns).

Secondary outcomes:

- Proportion of postnatal mothers who identified having a danger sign;
- Proportion of newborns with a danger sign identified; and
- Among those with an identified danger sign, the proportion of mothers and newborns who sought care from a health provider

### Data management

Data were collected electronically using tablets loaded with SurveyCTO. Data from the field were synchronized daily to the central cloud server. Daily checks were conducted on completeness and consistency of the collected data. The electronic data capturing system had limited data inaccuracies by built in logic, range and skip-patterns. Data were cleaned before the main analysis.

### Data analysis

Among all participating mothers, the proportion of mothers and newborns who have a postnatal check at the three time points were computed. The first comparative contrast was the proportion of mothers who had a postnatal check within 24 hours, 24–72 hours, and 73 hours to seven days between the pre- and post-intervention periods using a chi-square test. Absolute differences in the proportions, along with the corresponding 95% confidence intervales, are presented. Further descriptive analyses of differences in the recognition of and care-seeking for any postnatal maternal or neonatal danger signs and symptoms was conducted to demonstrate FPNC’s secondary effects. STATA version 15 software was used to analyze the data [26].

## Results

In the pre-intervention survey, a total of 144 mothers were screened, of which 119 met eligibility criteria and consented to participate in the study. After the introduction of FPNC (post-intervention phase) 133 mothers were screened, of which 115 met the eligibility criteria and 110 consented to participate in the study and took the checklist home. Of the 110 mothers who took the checklist home, 105 had family members collect the homecare kit (Fig 2).

**Fig 2:**
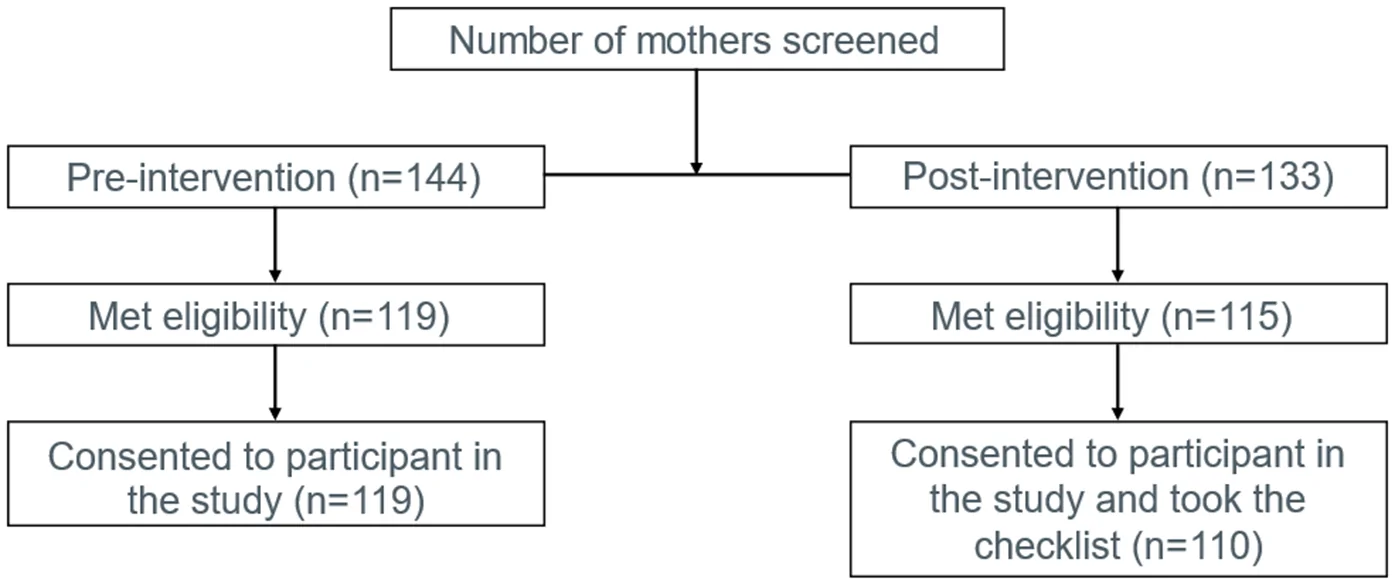
Participants flow diagram, Ada’a district, Oromia region of Ethiopia, 2023.

During both the survey periods, approximately 40% were between 18–24 years of age and around 45% were between 25–34 years of age (Table 1). Mothers’ distribution of the educational level, religion, and martial status were comparable between the two time periods. Approximately a quarter of mothers had no formal education and another half had only a primary level education. Ethiopian Orthodox was the predominant religion (>85%). More than 90% of the respondents in both pre- and post-intervention surveys were either currently married or cohabitating.

**Table 1:** Background characteristics of mothers included in the pre- (November 2022 – January 2023) and post-intervention periods (March-April 2023), Ada’a district, Oromia region of Ethiopia.

	Pre-intervention N = 119	Post-intervention N = 110	P-value
	n (%)	n (%)	
**Age in years**			
15-17	3 (3)	1 (1)	0.6871
18 - 24	48 (40)	44 (40)	
25- 34	55 (46)	49 (45)	
≥ 35	13 (11)	16 (15)	
**Education level**			
None	29 (24)	26 (23)	0.9277
Primary	64 (54)	58 (53)	
Secondary	24 (20)	25 (23)	
University	2 (2)	1 (1)	
**Religion**			
Orthodox	102 (867)	96 (87)	0.6928
Protestant	16 (13)	12 (11)	
Muslim	1 (1)	2 (2)	
**Marital status**			
Never married	6 (5)	5 (5)	0.7688
Currently married	89 (75)	87 (79)	
Separated	4 (3)	1 (1)	
Refused to answer	1 (1)	1 (1)	
Cohabitating	19 (16)	16 (14)	

### Coverage of postnatal checks within 24 hours, 24 – 72 hours, and 73 hours -seven days

As shown in Table 2 and Table 3, in the first 24 hours after birth, 97% of the mothers and newborns had a postnatal check in the pre-intervention survey, while 100% of the mothers and newborns had a postnatal check in the post-intervention period (P = 0.053). In the pre-intervention survey, less than 11% of mothers and newborns had a check between 24 and 72 hours, and only 3% had a check between 73 hours and seven days postpartum. In the post-intervention period, 96% of mothers and newborns had a check between 24 and 72 hours and between 73 hours and seven days postpartum (P < 0.0001).

**Table 2:** Postnatal checks for mothers during the first week after birth in the pre (November 2022 – January 2023) and post-intervention periods (March-April 2023), Ada’a district, Oromia region of Ethiopia.

Timing of postnatal checks	Mothers					
	Pre-intervention N = 119	Post-intervention N = 110	Absolute differences in %	95% confidence intervals	P value	X^2^
	n (%)	n (%)				
Within 24 hours	115 (96.6%)	110 (100%)	3.4%	(0.12%, 6.6%)	0.053	3.6
24 to 72 hours	11 (9.2%)	105 (95.5%)	86.3%	(79.7%, 92.7%)	<0.0001	169.2
73 hours to seven days	3 (2.5%)	105 (95.5%)	93%	(88.2%, 97.7%	<0.0001	197.2

**Table 3:** Postnatal checks for newborns during the first week after birth in the pre (November 2022 – January 2023) and post-intervention periods (March-April 2023), Ada’a district, Oromia region of Ethiopia.

Timing of postnatal checks	Newborns					
	Pre-intervention N = 119	Post-intervention N = 110	Absolute differences in %	95% confidence intervals	P value	X^2^
	n (%)	n (%)				
Within 24 hours	115 (96.6%)	110 (100%)	3.4%	(0.12%, 6.6%)	0.053	3.8
24 to 72 hours	13 (10.9%)	105 (95.5%)	84.6%	(77.7%,91.4%)	<0.0001	162.8
73 hours to seven days	3 (2.5%)	105 (95.5%)	93%	(88.2%, 97.7%	<0.0001	197.2

### Assessment of postnatal signs and symptoms

During the pre-intervention period, in the first 24 hours after birth, mothers reported being checked for general headache (57%), breast condition (53%), bleeding (48%), blood pressure (42%), temperature (39%), and face and leg swelling (9%). Post intervention, checklists revealed that mothers and families conducted self-checks at home for postnatal danger signs and symptoms, which resulted in nearly all women having the above-mentioned checks. Pre-intervention, from 24 hours to 72 hours and 73 hours to seven days, less than 10% of mothers had these signs and symptoms checked, while post-intervention over 95% had all of the signs and symptoms checked during the specified time period (Fig 3). This change is statistically significant (P < 0.0001).

**Fig 3:**
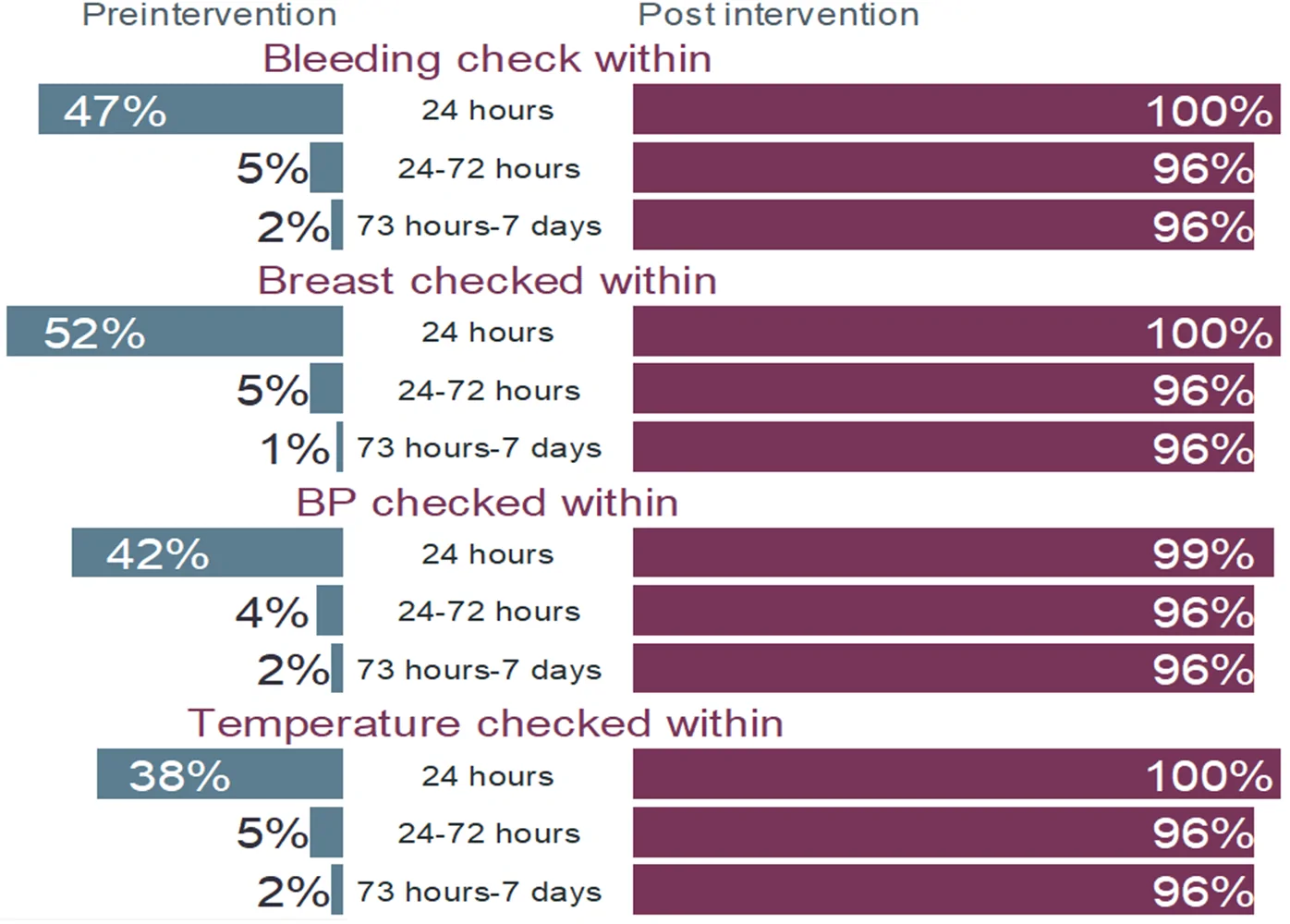
Maternal signs and symptoms checked during the first week after birth in the pre (November 2022 – January 2023) and post-intervention periods (March-April 2023), Ada’a district, Oromia region of Ethiopia.

For newborns, during the pre-intervention period, in the first 24 hours after birth mothers reported checks for breastfeeding (79%), umbilical cord (79%), general wellbeing (68%), breathing pattern (47%), temperature (36%), and skin color (25%). During the post-intervention period, almost all newborns had all the above-mentioned checks in the first 24 hours after birth. Pre-intervention, from 24 hours to 7 days, less than 10% of newborns had these signs and symptoms checked, while post-intervention over 95% (P < 0.0001) had all of the signs and symptoms checked in the same time period (Fig 4).

**Fig 4:**
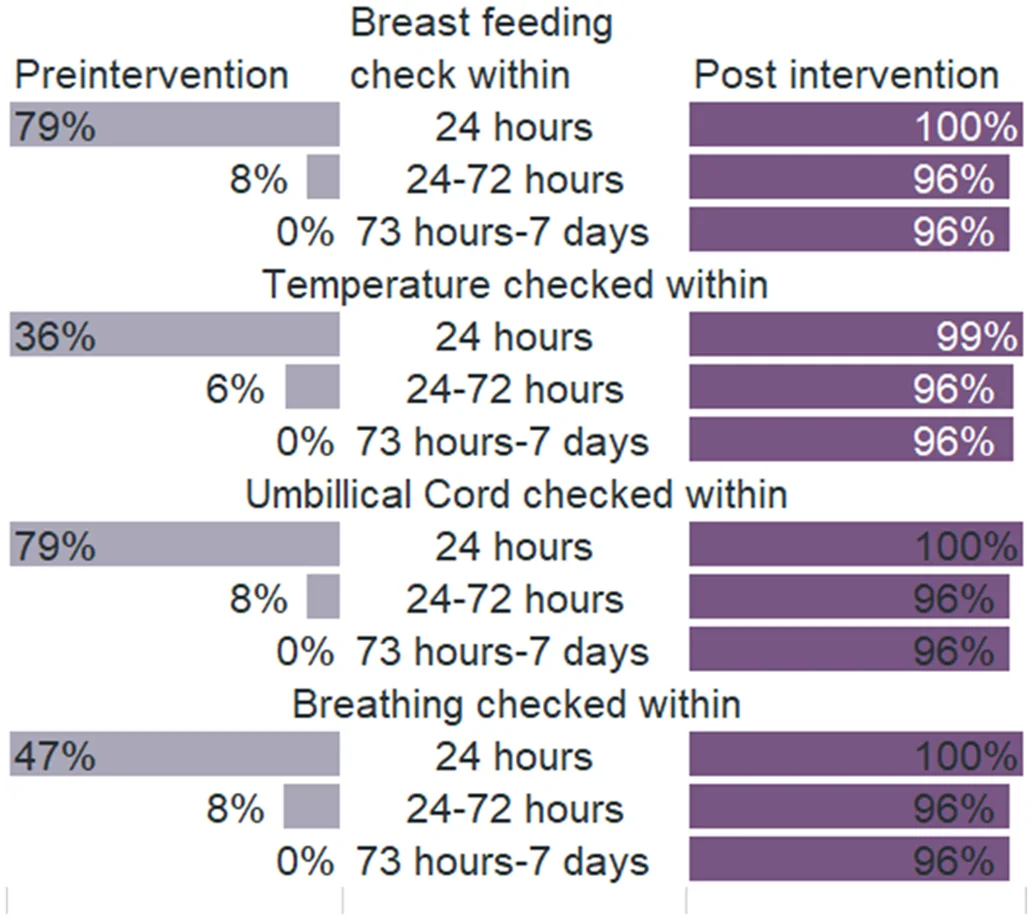
Newborn signs and symptoms checked during the first week after birth in the pre (November 2022 – January 2023) and post-intervention periods (March-April 2023), Ada’a district, Oromia region of Ethiopia.

### Detecting and seeking care for danger signs

As shown in Table 4, pre-intervention, 7% of mothers detected a danger sign, while post-intervention,18% detected a danger sign (P = 0.008). Among those who detected a danger sign, 63% in the pre-intervention period sought care (all from health centers), while in the post-intervention period, 80% sought care (65% from health centers and 15% health posts) (P = 0.4). During pre-intervention period, mothers detected headache (3%), bleeding (2%), breast conditions (2%), blood pressure problem (2%) and temperature (1%). Post-intervention, beyond identifying high blood pressure problem (11%), headache (5%), breast conditions (5%), and vaginal bleeding (4%), mothers additionally detected temperature problem (3%) and swollen face and leg (2%) as danger sign and symptoms (S1 Fig).

**Table 4:** Detected danger sign and care seeking during the first week after birth among mothers and newborns in the pre (November 2022 – January 2023) and post-intervention (March-April 2023) periods, Ada’a district, Oromia region of Ethiopia.

Variables	Mothers				Newborns			
	Pre-intervention N= 119	Post-intervention N= 110	P value	X^2^	Pre-intervention N= 119	Post-intervention N= 110	P value	X^2^
	n (%)	n (%)			n (%)	n (%)		
Detected one or more danger signs	8 (7)	20 (18)	0.008	6.96	5(4)	3 (3)	0.5	0.4
Among those with a danger sign detected	**n=8**	**n=20**			**n=5**	**n=3**		
Sought care from a provider	5 (63)	16 (80)	0.4	0.90	4 (80)	3 (100)	0.4	0.6

Among newborns, pre-intervention, a danger sign was detected in 4%, while post-intervention a danger sign was detected in 3% (P = 0.5). Among families who detected a danger sign, 80% sought care (all from health centers) pre-intervention, while 100% sought care (33% from health post and 67% from health center) post-intervention (P = 0.4) (Table 4). In the newborn, pre-intervention, breastfeeding problems (3%), umbilical cord conditions (3%), temperature problems (1%), and problems with breathing pattern (2%) were the signs and symptoms detected. In the post-intervention period, umbilical cord conditions (2%) and skin problems (1%) were the detected danger signs and symptoms (S2 Fig).

Compared to pre– intervention, the post-intervention survey showed an increase in the proportion of mothers who reported having knowledge on how to detect a problem in themselves (83% vs 97%, p < 0.001) and their newborns (92% vs 100% p = 0.003). Similarly, an increase in knowledge was noted on what actions to take for a problem detected in themselves (88% vs 97%, p = 0.009). No change was seen for actions to take for a newborn problem (98% vs 99%, p = 0.67) (Table 5).

**Table 5:** Mothers perception of her ability to detect health problem and her knowledge of actions to take in the pre (November 2022 – January 2023) and post-intervention (March-April 2023) periods, Ada’a district, Oromia region of Ethiopia.

	Pre-intervention N=119	Post-intervention N=110	P-value	X^2^
	n (%)	n (%)		
Mother knows:				
How to detect she has a problem	99 (83)	107 (97)	<0.001	12.5
What actions she should take if there is a maternal problem	105 (88)	107 (97)	0.009	6.8
How to detect that her newborn has a problem	110 (92)	110 (100)	0.003	8.6
What actions she should take if there is a newborn problem	117 (98)	109 (99)	0.67	0.3

## Discussion

This study aimed to assess if a family-led model for carrying out postnatal checks could increase the coverage of postnatal checks during the first week after birth. Nearly all mothers during pre- and post- intervention periods had postnatal checks in the 24 hours after birth. Pre-intervention, one in ten mothers and newborns had a postnatal check between 24 and 72 hours after birth, whereas during the post-intervention period, nine in ten mothers and newborns had a postnatal check. There was a similar increase in the proportion of mothers and newborns having postnatal checks between 73 and seven days. Results from this study suggest that mothers supported by their families, doing and documenting their home-based checks led to the increased number of postnatal checks in the post-intervention phase. Compared to pre-intervention, a larger proportion of mothers detected a maternal danger sign during the post-intervention period (7% vs. 18%) and sought care (63% vs. 80%). In contrast, very few families detected a newborn danger sign in either pre- or post- (4% vs. 3%) intervention periods and nearly all sought care (80% vs. 100%).

Completion of postnatal checks in the first 24 hours was high for mothers and newborns during the pre- and post-intervention periods, with almost all reporting completion of a check. This contrasts with data from the EDHS 2016, which showed that less than two in ten mothers and newborns received a check in the first 48 hours [12]. The difference in findings between the two studies may be because our data collection was limited to mothers having facility deliveries, while EDHS included both home and facility deliveries. The FPNC intervention did not appear to impact completion of postnatal checks in the first 24 hours, likely because the model is initiated at the facility level where most if not all mothers who give birth have at least one postnatal check prior to discharge.

Despite high coverage of postnatal check in the first 24 hours in both pre- and post-intervention periods, the quality of the checks provided by the health center staff was improved in the post-intervention period. Per the country guidelines, PNC checks for mothers should assess signs and symptoms of bleeding, pre-eclampsia, and sepsis, amongst others. Pre-intervention, only four in ten mothers were checked for bleeding, blood pressure and temperature. Although low, this proportion was much higher than reported in another study [16]. In contrast, in the post-intervention period almost all mothers had their uterine bleeding, blood pressure, and temperature checked.

Similarly, pre-intervention in the first 24 hours, eight in 10 newborns had their cord checked and were assessed for breastfeeding, and one in three had their temperature checked. This was much higher than what was reported in the Mini-EDHS 2019 for cord check (26%) and breastfeeding assessment (34%), while temperature measurement was comparable (26%) [27]. After the FPNC intervention was implemented, almost all newborns had all recommended components of the postnatal checks completed. The increased comprehensiveness in the post-intervention period can likely be attributed to the use of checklists by the health center staff during discharge. These findings suggest that systematic and improved discharge counselling process for postnatal mother and family and integrating a checklist as a tracking tool may have increased the quality and comprehensiveness of postnatal care. Family involvement in discharge counseling was an integral component of the FPNC intervention and may have contributed to improved postnatal care practices. However, the present study did not directly assess continued family support at home, and therefore the contribution of this component cannot be determined.

Per national guidelines, health extension workers are expected to provide home visits on the third and seventh day. Yet, pre-intervention, very few mothers and newborns received the recommended number of postnatal checks between 24 hours and 7 days. These findings are similar to those of a study that showed about one in ten mothers who delivered in a facility received a postnatal check between 24 hours and seven days after delivery [16]. Post-intervention, 96% of families retrieved the homecare kit and almost all mothers and newborns received postnatal checks between 24 hours and seven days. Thus, the homecare kit, checklist, and family engagement may have served as key resources and reminders for the mothers and families to assess key maternal and neonatal signs and symptoms. In the event that a mother delivers at home, has heard about the HCK, and a family member arrives to retrieve the HCK, the custodian will have extra checklists available to give to the family member. Thereby, decreasing the chance of the mothers not getting a postnatal check. But those mothers who gave birth at home were not included in the current analysis. Future studies can also investigate if this model also works for mothers who are unable to deliver in health facilities.

In this study, pre-intervention 7% of mothers detected a danger sign of which 63% sought care from a provider. Even though fewer mothers detected a danger sign in our study in the pre- intervention period (7% vs 16%), the proportion of care seeking was higher than a study in Kenya, where only 28% of postpartum mothers with a danger sign sought care [28]. After the intervention, the proportion of mothers who detected a danger sign increased (18%) and a majority of them (80%) sought care. This is similar to reports from previous studies where mothers assessed themselves based on a text message sent to them in the postpartum period [28,29]. Use of the text messages in these studies and engagement of families served as reminders to conduct health checks and consequently seek care when detecting danger signs. These findings suggest that it is possible to directly engage mothers and influence care-seeking using this innovative self-care model. For newborns in both the pre- and post-intervention surveys, very few detected a danger sign and almost all sought care. The number of families in this study who detected a danger sign is lower in both time periods as compared to the aforementioned Kenya study [28]. This can be attributed to the difference in the data collection period, where the other study collected data on danger signs in a two-month period after birth as compared to the one-week period used in this study and different study areas. For mothers and newborns, pre-intervention, those who sought care did so from the health center while post intervention, some sought care from health posts as well. In this study, the findings regarding danger sign detection and care-seeking should be interpreted in light of the small number of events observed, which limits the strength of the evidence and the certainty of their clinical relevance.

The feasibility and acceptability of the FPNC model have been demonstrated in a complementary mixed-methods study, which found that the model was acceptable to families and health workers, feasible to implement using simple pictorial tools and existing health-system structures, and supportive of linkage with formal health services. These findings provide context for the observed changes in postnatal outcomes and support the potential sustainability and scalability of the model, while recognizing that further evaluation of implementation costs, long-term sustainability, and safety is warranted [30].

This study has several notable strengths, particularly the use of a locally co-designed and led intervention created to be compatible with community cultural practices and beliefs, which was evaluated in a real-world setting. The intervention leveraged multiple key priorities of local government with regard to improving coverage of PNC and increasing access to self-care interventions. However, several limitations should be noted. First, this pilot study did not randomize participants to intervention versus control. Use of a pre-/post- design creates the possibility that effects were due to period-related influences other than the study intervention; however, we are unaware of other interventions or time-based factors that could have contributed to the observed changes in study measures. The study’s reliance on self-report for some measures, including aspects of checklist completion, may have been impacted by social desirability bias. The primary outcomes were assessed using maternal recall in the pre-intervention survey, whereas the post-intervention survey used a standardized home monitoring checklist completed by women and their family members. Although the checklist likely improved the accuracy of outcome ascertainment, this difference in measurement approach should be considered when interpreting comparisons between the two surveys. The Hawthorne effect could have contributed to the observed difference, as participants enrolled in the post-intervention period who were provided with a checklist may have assumed or known that they would be asked about completion of postnatal checks. The number of participants enrolled was relatively small, though the study is powered; thus, generalizability is likely to be limited. We included four health centers in the study; therefore, meaningful between facility variation was not anticipated, and clustering was not incorporated into the analysis.

In conclusion, FPNC is a promising self-care model that has the potential to improve home-based assessment and facilitate earlier identification of maternal and newborn complications, particularly among families who may otherwise have limited access to postnatal care. However, improvements in home assessment and recognition of complications do not necessarily translate into timely and appropriate interventions by health care providers or improved quality of postnatal care. Further evaluation using more rigorous designs, such as a cluster-randomized trial, is warranted to assess the effectiveness of FPNC when integrated within a complete continuum of postnatal care and supported by timely linkage to appropriate health services..

## Supporting information

S1 Fig Maternal danger signs detecred during the first week after birth in the pre (November 2022 – January 2023) and post-intervention periods (March-April 2023), Ada’a district, Oromia region of Ethiopia. (TIF)

S2 Fig Newborn danger signs detecred during the first week after birth in the pre (November 2022 – January 2023) and post-intervention periods (March-April 2023), Ada’a district, Oromia region of Ethiopia. (TIF)

S1 File STROBE Checklist: STROBE Checklist. (DOCX)
