# Supplementary material for: Changes in postnatal outcomes following implementation of a family-led postnatal care model: A pre-post intervention study in Ada’a District, Ethiopia
Source: PLOS Glob Public Health. 2026 Oct 30;6(10):e0006487. doi: 10.1371/journal.pgph.0006487 (PMC13722183; doi:10.1371/journal.pgph.0006487)
Supplement: Supplementary file 2 [file pgph.0006487.s005.docx]

**Response to Reviewers**

**Manuscript: Changes in Postnatal Outcomes Following Implementation of a Family-Led Postnatal Care Model: A Pre-Post Intervention Study in the Ada’a District, Ethiopia**
**Date: August 23, 2026**

**Dear Editor and Reviewers,**

We sincerely thank the Editor and the Reviewers for their thoughtful, constructive, and valuable comments on our manuscript. We greatly appreciate the time and expertise invested in reviewing our work. We have carefully considered all comments and have revised the manuscript accordingly to improve its clarity, methodological rigor, interpretation, and overall quality.

In particular, we have revised the title to “Changes in Postnatal Outcomes Following Implementation of a Family-Led Postnatal Care Model: A Pre-Post Intervention Study in the Ada’a District, Ethiopia” and strengthened the interpretation of the findings to ensure that the conclusions remain consistent with the pre-post study design and the available evidence. We have also clarified the distinction between improvements in family-led home assessment and the broader quality of postnatal care, while incorporating evidence from our complementary mixed-methods study on the feasibility, acceptability, and sustainability of the family-led postnatal care model.

Below, we provide a detailed, point-by-point response to each reviewer comment. For each comment, we present: (1) the reviewer’s comment in full; (2) our response explaining how we addressed the comment; and (3) the specific changes made to the manuscript, including page and line numbers where applicable.

All revisions have been incorporated into the revised manuscript and are reflected in the tracked-changes version submitted with this response. A clean version of the revised manuscript is also provided for the Editor and Reviewers’ convenience.

We hope that the revisions adequately address the reviewers’ concerns and have strengthened the manuscript.

| **Journal Requirements** | | |
| --- | --- | --- |
| **S.N.** | **Comments** | **Response** |
| 1. | In the ethics statement in the Methods, you have specified that verbal consent was obtained. Please provide additional details regarding how this consent was documented and witnessed, and state whether this was approved by the IRB. | Line 222-223 The witness of the data collectors was recorded as their signature in the consent form. This procedure has been approved by IRB. |
| 2. | We noticed that you used “data not shown” in the manuscript. We do not allow these references, as the PLOS data access policy requires that all data be either published with the manuscript or made available in a publicly accessible database. Please amend the supplementary material to include the referenced data or remove the references. | Line 296-include the table as supplementary file |
| 3. | Fig 1: Please confirm whether you drew the images / clip-art within the figure panels by hand. If you did not draw the images, please provide (a) a link to the source of the images or icons and their license / terms of use; or (b) written permission from the copyright holder to publish the images or icons under our CC-BY 4.0 license. Alternatively, you may replace the images with open-source alternatives. See these open-source resources you may use to replace images / clip-art:  - [https://commons.wikimedia.org](https://track.editorialmanager.com/CL0/https:%2F%2Fcommons.wikimedia.org/1/010f019efd7c4bd6-1a7bf097-7fb8-4a9b-87ab-df3fef25c117-000000/2S1zJb41bqBcE7gm1i-ZDeKdpBf6yE4HkOeas21PgbE=258)  - [https://openclipart.org/](https://track.editorialmanager.com/CL0/https:%2F%2Fopenclipart.org%2F/1/010f019efd7c4bd6-1a7bf097-7fb8-4a9b-87ab-df3fef25c117-000000/j3oEqlnKCHRpq9675EM0etE66a2eSxscJASSWYN1FiI=258) | The graphic has been updated using all images sourced from openclipart.org and/or drawn by hand in Powerpoint using shapes by one of the project team. |
| 4. | Please provide separate figure files in .tif or .eps format | . Figures are provided separately using the suggested file format. |

| **Additional Editor Comments (if provided):** | | |
| --- | --- | --- |
| **S.N.** | **Comments** | **Response** |
| 1. | The title, abstract, and conclusion should avoid strong causal language such as “effectiveness” and “significantly increased,” given the uncontrolled pre and post design | In addition to excluding the significantly, we also edit the title and replace effectiveness by change in outcome “Changes in Postnatal Outcomes Following Implementation of a Family-Led Postnatal Care Model: A Pre-Post Intervention Study in the Ada'a District, Ethiopia “ |
| 2. | • The authors must clearly acknowledge that observed changes may reflect secular trends, selection bias, Hawthorne effects, measurement differences, or implementation intensity, not necessarily intervention effect alone | Thank you for the comments, we have incorporated to limitation. |
| 3. | The Methods section needs clearer reporting of recruitment, eligibility, screening losses, consent, sampling, timing of surveys, and comparability of pre and post groups. | Thank you for the important feedback. The methods section is elaborated now. The pre and post intervention groups are comparable, as there is no significant difference between the two groups in terms of the background characteristics |
| 4. | Outcome measurement requires clarification, especially because pre intervention data appear largely based on recall, while post intervention outcomes rely on family completed checklists | We have indicated both the absolute differences and the 95% CI for the primary outcome. |
| 5. | The authors should clarify whether clustering by health center was considered, and if not, explicitly discuss this as a limitation.: | Thank you for the comments, we have indicated both the absolute differences and the 95% CI for the primary outcome. |
| 6. | Results should be checked carefully for internal consistency, table numbering errors, denominator clarity, and typographical issues. | Thank you for the comments, since we have only four health centers, we do not expect variation among the four HCs and did not consider clustering. |
| 7. | Results should be checked carefully for internal consistency, table numbering errors, denominator clarity, and typographical issues. . | Thank you for this comment, we have clarified denominators for each table, adjusted table numbering errors and typographical issues which we show in track changes. |
| 8. | Interpretation of danger sign detection and care seeking should be cautious, as event numbers are small and clinical implications remain uncertain. | Comment well taken and have indicated it in the discussion section. |
| 9. | The Discussion should better address feasibility, safety, family capacity, device use, sustainability, cost, scalability, and linkage with formal health services | Comment well taken and have indicated it in the discussion section |
| 10. | The conclusion should be reframed to state that the model is promising and warrants more rigorous evaluation using a concurrent comparison group or cluster randomized design. | Comment well taken and have added it in the conclusion section |

| **Reviewers' comments:** | | |
| --- | --- | --- |
| **S.N.** | **Comments** | **Response** |
| 1. | Reviewer #1: I recommend that the authors avoid unnecessary details in each section, especially in the methods section.  The interventions applied to the study participants are not clearly indicated. What does family-led postnatal care mean? Which family members were primarily involved? Do these family members take actions or give care or simply report danger signs to the local health facilities? How were the family members trained? Who trained them? How about the self care? For example if the mother herself detects any danger sign, is that still included under the family led postnatal care? Please make all these clear | Thank you for the comments, these information are included in the project description Line number 108 under the introduction section and the paper to the full protocol is cited too |
| 2. | Reviewer #2: Effectiveness of family-led postnatal care model: a pre-post intervention study in the Ada'a District, Ethiopia Manuscript Review  Introduction The study speaks to a key area of concern in maternal and newborn health, rightly brought out by the authors, as poorly performing despite being a critical point of clinical intervention. In light of the new self-care guidelines provided, the family-led postnatal care model presents a novel look on how to incorporate the same to post-natal care. The intervention described focuses on a model that utilizes home-based interventions in the post-natal period to assess key indices in a postnatal mother or the newborn. The topic therefore needs to be reworded appropriately to reflect that the effectiveness is not on the postnatal care as alluded to (19), but rather on the effectiveness of the intervention to improve daily check of key indices as clearly described in 28-29. This will align with the referenced WHO recommendations (421)- | Thank you for the comments, we have changed topic to “Changes in Postnatal Outcomes Following Implementation of a Family-Led Postnatal Care Model: A Pre-Post Intervention Study in the Ada’a District, Ethiopia“ |
| 3. | Research aims The introduction notes that the objective of the study was to evaluate the effectiveness of family-led postnatal care intervention on coverage of postnatal check within seven days of delivery for both the mothers and newborns. (102-104). The authors reference seek to establish the effectiveness of a model that only addresses part of the definition of post-natal care as self-care does not involve the health workers. The research would best be framed to evaluate the effectiveness of home-based family-led post-natal care interventions in improving timely diagnosis of postnatal complications.: | Comment well taken and revised objective to “The objective of the current study was to evaluate potential changes in selected postnatal care-related outcomes following implementation of a family-led postnatal care intervention on coverage of postnatal checks within seven days of delivery for both mothers and newborns” |
| 4. | Materials and Methods There is a clear description of the FPNC model as the intervention. The sample population of the post-intervention cross-sectional survey is well described. The pre-intervention sample population is described in terms of sample size and population. However, the recruitment process into the study remains unclear as to confounding was accounted for if the discharge process was already deviated from the norm by the additional interventions by the discharging midwife. This affects the validity of the findings. | We thank the reviewer for this important comment. We would like to clarify that the discharge assessment and counseling were not an additional intervention but an integral component of the Family-Led Postnatal Care (FPNC) model (Figure 1). The FPNC intervention was designed as a package comprising the standardized discharge orientation followed by family-led home postnatal care. Therefore, any observed changes in outcomes reflect the effect of the complete FPNC model rather than the home-based component alone. The discharge counseling cannot be considered a confounder because it is part of the intervention being evaluated, not an external factor that varied independently of the intervention. Accordingly, it does not compromise the internal validity of the study findings. |
| 5. | Results and discussion The findings of the primary outcomes for the pre-intervention group relies on recall, does not establish the measure for the post-natal checks, whether health worker initiated or self-initiated, for a control group that has no exposure to self-care, introducing a bias at the point of data analysis. Further, the authors allude to the likelihood that involvement of the family members in the discharge counselling likely contributed to better follow up of the postnatal mother and newborn (349-351), but there is no data collected that demonstrates continued support of family members at home, and further, no comparison with the pre-intervention survey | We thank the reviewer for this important comment. We acknowledge that the primary outcomes were measured differently in the two surveys. In the pre-intervention survey, women reported postnatal checks based on recall using a structured questionnaire. In the post-intervention survey, women and their family members were provided with a standardized daily checklist to document postnatal checks performed from day 2 to day 7 following discharge. The checklist was introduced as part of the FPNC intervention to support home-based monitoring and to improve the completeness and accuracy of recording postnatal checks. We recognize that this difference in outcome measurement may have reduced recall bias in the post-intervention group and have acknowledged this as a study limitation in the revised manuscript. We have indicated this as one limitation of the study.  Regarding the Discussion, we agree that our statement on the contribution of family involvement was speculative. Although family participation in discharge counseling was an integral component of the FPNC intervention, we did not collect data on continued family support at home or compare family involvement between the pre- and post-intervention surveys. Accordingly, we have revised the discussion to avoid attributing the observed improvements to this specific mechanism and instead state that family engagement may have contributed to the intervention's effects, but this was not directly evaluated in the present study. Conclusion |
| 6. | Conclusion The conclusion requires to be restructured to reflect that the model does indeed improve objective home assessment and earlier diagnosis of complications, but without tying this to timely and appropriate interventions by the health care providers, then it is to possible to conclude on improved quality of PNC (396-398). However, it does rightly state that with rigorous design, the model can be better evaluated within a complete care system, on its effectiveness in strengthening PNC | Thank you for the comment, we have restructured the conclusion accordingly. |
