# Supplementary material for: Changes in postnatal outcomes following implementation of a family-led postnatal care model: A pre-post intervention study in Ada’a District, Ethiopia
Source: PLOS Glob Public Health. 2026 Oct 30;6(10):e0006487. doi: 10.1371/journal.pgph.0006487 (PMC13722183; doi:10.1371/journal.pgph.0006487)
Supplement: Peer review history

# Peer review: Decision Letter 0

**DOI:** 10.1371/journal.pgph.0006487.r001

**Authors:** Shiyam Sunder

25 Jun 2026

PGPH-D-26-01622

Effectiveness of family-led postnatal care model: a pre-post intervention study in the Ada’a District, Ethiopia

PLOS Global Public Health

Dear Dr. Regassa,

Thank you for submitting your manuscript to PLOS Global Public Health. After careful consideration, we feel that it has merit but does not fully meet PLOS Global Public Health’s publication criteria as it currently stands. Therefore, we invite you to submit a revised version of the manuscript that addresses the points raised during the review process.

Please submit your revised manuscript by Aug 24 2026 11:59PM. If you will need more time than this to complete your revisions, please reply to this message or contact the journal office at globalpubhealth@plos.org. When you're ready to submit your revision, log on to https://www.editorialmanager.com/pgph/ and select the 'Submissions Needing Revision' folder to locate your manuscript file.

Please include the following items when submitting your revised manuscript:

- A letter that responds to each point raised by the editor and reviewer(s). You should upload this letter as a separate file labeled 'Response to Reviewers'.
- A marked-up copy of your manuscript that highlights changes made to the original version. You should upload this as a separate file labeled 'Revised Manuscript with Track Changes'.
- An unmarked version of your revised paper without tracked changes. You should upload this as a separate file labeled 'Manuscript'.

Guidelines for resubmitting your figure files are available below the reviewer comments at the end of this letter.

As the corresponding author, your ORCID iD is verified in the submission system and will appear in the published article. PLOS supports the use of ORCID, and we encourage all coauthors to register for an ORCID iD and use it as well. Please encourage your coauthors to verify their ORCID iD within the submission system before final acceptance, as unverified ORCID iDs will not appear in the published article. *Only*  the individual author can complete the verification step; PLOS staff *cannot*  verify ORCID iDs on behalf of authors.

We look forward to receiving your revised manuscript.

Kind regards,

Shiyam Sunder, MBBS, MSc epidemiology, PhD

Academic Editor

PLOS Global Public Health

Journal Requirements:

1. In the ethics statement in the Methods, you have specified that verbal consent was obtained. Please provide additional details regarding how this consent was documented and witnessed, and state whether this was approved by the IRB.

1. We noticed that you used “data not shown” in the manuscript. We do not allow these references, as the PLOS data access policy requires that all data be either published with the manuscript or made available in a publicly accessible database. Please amend the supplementary material to include the referenced data or remove the references.

1. Fig 1: Please confirm whether you drew the images / clip-art within the figure panels by hand. If you did not draw the images, please provide (a) a link to the source of the images or icons and their license / terms of use; or (b) written permission from the copyright holder to publish the images or icons under our CC-BY 4.0 license. Alternatively, you may replace the images with open-source alternatives. See these open-source resources you may use to replace images / clip-art:

- https://commons.wikimedia.org

- https://openclipart.org/

1. Please provide separate figure files in .tif or .eps format.
  For more information about figure files please see our guidelines:
  https://journals.plos.org/globalpublichealth/s/figures
  https://journals.plos.org/globalpublichealth/s/figures#loc-file-requirements

If the reviewer comments include a recommendation to cite specific previously published works, please review and evaluate these publications to determine whether they are relevant and should be cited. There is no requirement to cite these works unless the editor has indicated otherwise.

Additional Editor Comments (if provided):

The manuscript addresses an important postnatal care gap and presents a promising family led model, but substantial revision is required before further consideration.

• The title, abstract, and conclusion should avoid strong causal language such as “effectiveness” and “significantly increased,” given the uncontrolled pre and post design.

• The authors must clearly acknowledge that observed changes may reflect secular trends, selection bias, Hawthorne effects, measurement differences, or implementation intensity, not necessarily intervention effect alone.

• The Methods section needs clearer reporting of recruitment, eligibility, screening losses, consent, sampling, timing of surveys, and comparability of pre and post groups.

• Outcome measurement requires clarification, especially because pre intervention data appear largely based on recall, while post intervention outcomes rely on family completed checklists.

• The analysis should report absolute differences and 95% confidence intervals, not only p values.

• The authors should clarify whether clustering by health center was considered, and if not, explicitly discuss this as a limitation.

• Results should be checked carefully for internal consistency, table numbering errors, denominator clarity, and typographical issues.

• Interpretation of danger sign detection and care seeking should be cautious, as event numbers are small and clinical implications remain uncertain.

• The Discussion should better address feasibility, safety, family capacity, device use, sustainability, cost, scalability, and linkage with formal health services.

• The conclusion should be reframed to state that the model is promising and warrants more rigorous evaluation using a concurrent comparison group or cluster randomized design.

[Note: HTML markup is below. Please do not edit.]

Reviewers' comments:

Reviewer's Responses to Questions

**Comments to the Author**

1. Does this manuscript meet PLOS Global Public Health’s publication criteria?

Reviewer #1: Partly

Reviewer #2: Partly

**********

2. Has the statistical analysis been performed appropriately and rigorously?

Reviewer #1: No

Reviewer #2: N/A

**********

3. Have the authors made all data underlying the findings in their manuscript fully available (please refer to the Data Availability Statement at the start of the manuscript PDF file)?

The PLOS Data policy

Reviewer #1: Yes

Reviewer #2: Yes

**********

4. Is the manuscript presented in an intelligible fashion and written in standard English?

Reviewer #1: Yes

Reviewer #2: Yes

**********

Reviewer #1: I recommend that the authors avoid unnecessary details in each section, especially in the methods section.

The interventions applied to the study participants are not clearly indicated.

What does family-led postnatal care mean? Which family members were primarily involved? Do these family members take actions or give care or simply report danger signs to the local health facilities? How were the family members trained? Who trained them? How about the self care? For example if the mother herself detects any danger sign, is that still included under the family led postnatal care? Please make all these clear.

Reviewer #2: Effectiveness of family-led postnatal care model: a pre-post intervention study in the Ada'a District, Ethiopia

Manuscript Review

Introduction

The study speaks to a key area of concern in maternal and newborn health, rightly brought out by the authors, as poorly performing despite being a critical point of clinical intervention. In light of the new self-care guidelines provided, the family-led postnatal care model presents a novel look on how to incorporate the same to post-natal care.

The intervention described focuses on a model that utilizes home-based interventions in the post-natal period to assess key indices in a postnatal mother or the newborn. The topic therefore needs to be reworded appropriately to reflect that the effectiveness is not on the postnatal care as alluded to (19), but rather on the effectiveness of the intervention to improve daily check of key indices as clearly described in 28-29. This will align with the referenced WHO recommendations (421)

Research aims

The introduction notes that the objective of the study was to evaluate the effectiveness of family-led postnatal care intervention on coverage of postnatal check within seven days of delivery for both the mothers and newborns. (102-104). The authors reference seek to establish the effectiveness of a model that only addresses part of the definition of post-natal care as self-care does not involve the health workers. The research would best be framed to evaluate the effectiveness of home-based family-led post-natal care interventions in improving timely diagnosis of postnatal complications.

Materials and Methods

There is a clear description of the FPNC model as the intervention. The sample population of the post-intervention cross-sectional survey is well described. The pre-intervention sample population is described in terms of sample size and population. However, the recruitment process into the study remains unclear as to confounding was accounted for if the discharge process was already deviated from the norm by the additional interventions by the discharging midwife. This affects the validity of the findings.

Results and discussion

The findings of the primary outcomes for the pre-intervention group relies on recall, does not establish the measure for the post-natal checks, whether health worker initiated or self-initiated, for a control group that has no exposure to self-care, introducing a bias at the point of data analysis. Further, the authors allude to the likelihood that involvement of the family members in the discharge counselling likely contributed to better follow up of the postnatal mother and newborn (349-351), but there is no data collected that demonstrates continued support of family members at home, and further, no comparison with the pre-intervention survey

Conclusion

The conclusion requires to be restructured to reflect that the model does indeed improve objective home assessment and earlier diagnosis of complications, but without tying this to timely and appropriate interventions by the health care providers, then it is to possible to conclude on improved quality of PNC (396-398). However, it does rightly state that with rigorous design, the model can be better evaluated within a complete care system, on its effectiveness in strengthening PNC.

**********

what does this mean?). If published, this will include your full peer review and any attached files.

**Do you want your identity to be public for this peer review?**  If you choose “no”, your identity will remain anonymous but your review may still be made public.

For information about this choice, including consent withdrawal, please see our Privacy Policy

Reviewer #1: No

Reviewer #2: **Yes:**  Nelly Bosire

**********

[NOTE: If reviewer comments were submitted as an attachment file, they will be attached to this email and accessible via the submission site. Please log into your account, locate the manuscript record, and check for the action link "View Attachments". If this link does not appear, there are no attachment files.]

# Author comment: Author response to Decision Letter 1

**DOI:** 10.1371/journal.pgph.0006487.r002

23 Aug 2026

Attachment Submitted filename: Response to Reviewers_FPNC.docx

# Editor report: Decision Letter 1

**DOI:** 10.1371/journal.pgph.0006487.r003

**Authors:** Shiyam Sunder

8 Oct 2026

Changes in Postnatal Outcomes Following Implementation of a Family-Led Postnatal Care Model: A Pre-Post Intervention Study in Ada'a District, Ethiopia

PGPH-D-26-01622R1

Dear Dr Regassa,

We are pleased to inform you that your manuscript 'Changes in Postnatal Outcomes Following Implementation of a Family-Led Postnatal Care Model: A Pre-Post Intervention Study in Ada'a District, Ethiopia ' has been provisionally accepted for publication in PLOS Global Public Health.

Before your manuscript can be formally accepted you will need to complete some formatting changes, which you will receive in a follow up email. A member of our team will be in touch with a set of requests.

Please note that your manuscript will not be scheduled for publication until you have made the required changes, so a swift response is appreciated.

IMPORTANT: The editorial review process is now complete. PLOS will only permit corrections to spelling, formatting or significant scientific errors from this point onwards. Requests for major changes, or any which affect the scientific understanding of your work, will cause delays to the publication date of your manuscript.

If your institution or institutions have a press office, please notify them about your upcoming paper to help maximize its impact. If they'll be preparing press materials, please inform our press team as soon as possible -- no later than 48 hours after receiving the formal acceptance. Your manuscript will remain under strict press embargo until 2 pm Eastern Time on the date of publication. For more information, please contact globalpubhealth@plos.org.

Thank you again for supporting Open Access publishing; we are looking forward to publishing your work in PLOS Global Public Health.

Best regards,

Shiyam Sunder, MBBS, MSc epidemiology, PhD

Academic Editor

PLOS Global Public Health

***********************************************************

Recommendation: Accept with minor editorial corrections

Thank you for submitting the revised manuscript and for carefully addressing the reviewers' comments.

The manuscript has improved substantially in its methodological reporting, presentation of findings, and interpretation. The revised title and conclusions are more consistent with the observational pre-post study design. The study addresses an important maternal and newborn health priority and provides useful preliminary evidence regarding the potential of family-led postnatal assessments in resource-constrained settings.

The authors have appropriately acknowledged key methodological limitations, including the absence of a concurrent comparison group and differences in outcome measurement between study periods.

I am satisfied with the revisions and consider the manuscript suitable for publication. I recommend acceptance, subject to minor editorial corrections and a final consistency check of the tables and statistical reporting.

Reviewer Comments (if any, and for reference):
